# Potential Role of Caveolin-1 in Regulating the Function of Endothelial Progenitor Cells from Experimental MODS Model

**DOI:** 10.1155/2019/8297391

**Published:** 2019-04-17

**Authors:** Tianhang Luo, Jixin Shu, Zhengmao Lu, Ting Han, Guoen Fang, Xuchao Xue

**Affiliations:** ^1^Department of General Surgery, Changhai Hospital, The Second Military Medical University, Shanghai 200433, China; ^2^Department of General Surgery, Gongli Hospital, The Second Military Medical University, Shanghai 200135, China

## Abstract

Multiple organ dysfunction syndrome (MODS) remains a great challenge in critical care because of its common occurrence, high cost of care, and high mortality. Vascular endothelial injury is the initiation step in the development of MODS, and EPCs are essential for the process of organ repair. It is unclear whether and how caveolin-1 (Cav-1) in EPCs contributes to the pathogenesis of MODS. The present study is aimed at investigating the potential role of Cav-1 in EPCs during MODS. We established a MODS model in pigs, isolated and characterized EPCs from the MODS model, and tracked Cav-1 expression and various in vitro behaviors of EPCs from the MODS model. Then, we knockdown Cav-1 expression with siRNA or induce Cav-1 expression with proinflammatory factors to evaluate potential effects on EPCs. Our results suggest that Cav-1 expression correlated with EPC functions during MODS and Cav-1 regulates the function of endothelial progenitor cells via PI3K/Akt/eNOS signaling during MODS. Thus, Cav-1 in EPCs could be an attractive target for the treatment of MODS.

## 1. Introduction

Multiple organ dysfunction syndrome (MODS), caused by severe sepsis, remains a great challenge in critical care because of its common occurrence, high cost of care, and high mortality. It is one of the major causes of death in intensive care unit (ICU) patients with a mortality rate of 14–60% in various reports [1]. In most patients, endothelial cells (ECs) not only participate in inflammatory reactions but also are the first damaged target cells. Vascular endothelial injury is the initiation step in microcirculation disturbance of important organs, and it might trigger the development of MODS [2]. Accumulating evidence suggests that vascular endothelial regeneration requires the mobilization, proliferation, and differentiation of bone marrow-derived endothelial progenitor cells (EPCs) [3].

EPCs, also known as angioblasts, are involved in angiogenesis at birth as well as vascular regeneration and repair of organ damage caused by trauma or severe sepsis. Regenerative cell therapy with EPCs may potentially offer a new modality to reestablish perfusion and restore the function of injured organs [4]. In our previous research, autologous transplantation of EPCs was applied to treat trauma-induced MODS, and the transplanted EPCs could migrate to injured organs and induce angiogenesis to restore blood flow which could improve the function of important organs. However, the mortality rate in the transplantation group (45%) remained higher than expected [5]. These findings indicated that the inflammatory environment might hinder the repair of vascular endothelial injury by EPCs during the progression of MODS. Therefore, understanding the molecular mechanism underlying EPC behaviors in MODS is essential for its treatment.

Caveolae, a subset of membrane (lipid) rafts, is a flask-like invagination of the plasma membrane in most cells [6]. Caveolin-1 (Cav-1), the main structural protein component of caveolae, plays an important role in maintaining its structure [7] and regulating signaling pathways involved in cell growth and differentiation [6]. Several studies reported that Cav-1 regulates the function of EPCs in inflammatory diseases. In endothelial colony-forming cells (ECFCs) that possess EPC features, caveolae-disrupting agents and caveolin-1 knockdown were employed to demonstrate that the angiogenic properties of ECFCs are associated with caveolae integrity [8]. In addition, Cav-1 suppresses neuronal differentiation by downregulating vascular endothelial growth factor (VEGF), p44/42MAPK, Akt, and Stat3 pathways [9]. High glucose levels inhibit the function of bone marrow-derived EPCs, likely by degrading the eNOS-caveolin-1 complex, resulting in altered function of EPCs [10]. Furthermore, high caveolin-1 levels mediate inflammatory breast cancer (IBC) cell invasion by activating Akt1, which in turn causes RhoC GTPase phosphorylation [11]. However, it is unclear that whether and how Cav-1 in EPCs contributes to the pathogenesis of MODS.

The present study is aimed at investigating the potential role of Cav-1 in EPCs during MODS. We established a MODS model in pigs and tracked Cav-1 expression and various in vitro behaviors of EPCs from the MODS model. Then, we knockdown Cav-1 expression with siRNA or induce Cav-1 expression with proinflammatory factors to evaluate potential effects on EPCs. Our results suggest that caveolin-1 regulates the function of endothelial progenitor cells via PI3K/Akt/eNOS signaling during MODS.

## 2. Materials and Methods

### 2.1. Animals

Assays involving animals were carried out according to the Chinese legislation on the protection of animals and the 1996 National Institutes of *Health Guide for the Care and Use of Laboratory Animals* [12]. The animal experiments were conducted at the Laboratory Animal Research Center, Second Military Medical University (Shanghai, China), in an Association for Assessment and Accreditation of Laboratory Animal Care-approved facility (No. 001003), after approval from the Institutional Animal Care and Use Committee.

A total of 30 male Banna pigs (22.41 ± 1.33 kg) were used in the present study. The animals were maintained at room temperature (20 to 25°C), with daylight and free access to tap water and standard food. Before the experiments, the pigs were fasted overnight but with free access to water for 24 h. Two animal groups were randomly assigned as the MODS (subjected to hemorrhagic shock, resuscitation, and endotoxemia; *n* = 20) and control (sham operation; *n* = 10) groups.

### 2.2. Anesthesia and Positioning

Anesthesia and positioning were performed as previously described [5]. Briefly, 15 mg/kg ketamine hydrochloride (Pfizer, Germany), 0.4 mg/kg diazepam (Sunrise, China), and 0.02 mg/kg atropine (Braun, Germany) were injected intramuscularly, followed by intravenous administration of 1 mg/kg etomidate (Braun) for anesthesia. Oral intubation was carried out before mechanical ventilation (Evita, Dräger, Lübeck, Germany). The animals were then placed in the lateral decubitus position, alternating their right and left sides for bilateral access. At experimental end, the survival animals were euthanized by an overdose of pentobarbital sodium (Tianyi, Xian, China) administered intravenously.

### 2.3. Operation

The two groups underwent the same surgical procedure under asepsis. First, the arteria carotis interna was dissected and intubated with a 12 G retention catheter (Arrow, Leeds, UK) to monitor arterial blood pressure. Next, the left femoral artery and femoral vein were intubated with an 8F Swan-Ganz catheter (Arrow) for the monitoring of pulmonary arterial pressure, pulmonary arterial wedge pressure, ventricular stroke output, and central venous pressure. A retention catheter was introduced into the right femoral artery for exsanguination. The skin was sutured postsurgery.

### 2.4. Hemorrhagic Shock, Resuscitation, and Endotoxemia

The MODS group was subjected to hemorrhagic shock, resuscitation, and endotoxemia postoperation. The hemorrhagic shock was induced by the modified Wiggers procedure as previously described [13, 14]. In brief, hemorrhagic shock was produced for 120 minutes by blood-letting via the femoral artery until the mean arterial blood pressure (MAP) reached 6.7 ± 0.67 kPa (50 ± 5 mmHg) in 30 minutes. The volume of lost blood to achieve this blood pressure varied among individual animals. Then, 60% volume of lost blood and lactated Ringer's solution (Otsuica, China) and twice volume of lost blood in 60 minutes were transfused through a central venous catheter. MAP must reach over 80% of the value before hemorrhagic shock. The pigs in the MODS group were intravenously injected with 0.5 mg/kg lipopolysaccharide of *E. coli* (E.colO111B4; Sigma, St. Louis, Missouri, USA) 12 hours after resuscitation for 24 hours. The control group was only transfused with 5% glucose solution post sham operation.

### 2.5. Organ Function Monitoring and Support

The two groups were subjected to electrocardiographic monitoring, with several indexes assessed, including MAP, breathing rate, heart rate, central venous pressure, pulmonary artery pressure, pulmonary artery wedge pressure, and cardiac output (CO). Then, serum alanine aminotransferase (ALT), aspartate aminotransferase (AST), total bilirubin (TB), creatinine (Cr), blood urea nitrogen (BUN), white blood cell count, blood platelet count, arterial oxygen saturation, arterial partial pressure of oxygen (PaO_2_), arterial partial pressure of carbon dioxide (PaCO_2_), and arterial potential of hydrogen were measured 24 hours preoperatively (T1) and at 12 hours after resuscitation (T2), 24 hours after endotoxemia (T3), 72 hours after endotoxemia (T4), 96 hours after endotoxemia (T5), 144 hours after endotoxemia (T6), and 168 hours after endotoxemia (T7).

Respiration and circulation in both groups were monitored continuously. At PaO_2_ < 60 mmHg or PaCO_2_ > 60 mmHg, artificial ventilation was initiated. With MAP less than two-thirds of the normal value, dopamine was administered. The animals were positioned in the lateral decubitus position, as described above. Metabolic support was achieved with a caloric intake rate twice the basic energy expenditure (104 ± 3.9 kcal/kg^0.75^/day in pigs), comprising amino acids, 10% fat emulsion, and glucose, administered intravenously. Daily protein intake was 1.5 g/kg, with a calorie-to-nitrogen ratio of 180 : 1. The animals received potassium 1.5 g per day and 100-150 mL/kg/day of water. Ketamine hydrochloride (5 mg/kg/day) and diazepam (0.2 mg/kg/day) were administered to maintain general anesthesia. All animals that survived were euthanized at 168 h after endotoxemia.

### 2.6. Diagnostic Criteria of MODS

According to related studies [15], the following diagnostic criteria for MODS were adopted: (1) pulmonary dysfunction (breathing rate > 40 per minute, PaO_2_ < 60 mmHg, or PaCO_2_ > 40 mmHg); (2) cardiac dysfunction (cardiac dysrhythmia, CO exceeding two times the upper limit of normal (ULN), heart rate < 60, or MAP < 70% of the ULN); (3) coagulation disorders (blood platelet count < 70% of the ULN or prothrombin time and thrombin time 3 seconds longer than the ULN); (4) hepatosis (serum ALT, aspartate aminotransferase, or TB more than two times of normal); and (5) renal dysfunction (serum Cr or BUN more than two times the ULN). The following upper limit of normal means (ULN) was adopted in our study: CO: 4.5 L/min, MAP: 110 mmHg, blood platelet count: 100^∗^109/L, prothrombin time: 11 s, ALT: 40 U/L, TB: 18 *μ*mol/L, Cr: 100 *μ*mol/L, and BUN: 9 mmol/L. MODS was diagnosed with two or more criteria met.

### 2.7. EPC Culture

Mononuclear cells were isolated by density-gradient centrifugation with Ficoll (1.077 g/mL; Sigma) from the bone marrow in animals of both groups at indicated time points, with ethylenediaminetetraacetic acid (EDTA) as an anticoagulant. After isolation, mononuclear cells were immediately seeded at a density of 1 × 10^5^/cm^2^ on 6-well culture dishes coated with 2% human fibronectin (Chemicon, Billerica, MA) and maintained in EPC growth medium 2 (EGM2, Promo Cell, Heidelberg, Germany) for 2 hours to reduce macrophage disturbance. Two hours after seeding, nonadherent cells were collected and reseeded; 4 days after culturing, nonadherent cells were removed by a thorough washing with phosphate-buffered saline (PBS), and adherent cells were cultured in fresh medium. Seven days after initiation of mononuclear cell culture, adherent cells were elongated into a spindle shape and grew into colonies of EPCs. The microstructure of EPCs was observed by electron microscopy.

### 2.8. Characterization of EPCs

The flow cytometry analysis of surface markers of EPCs, including CD133 (polyclonal goat CD133 antibody, Santa Cruz, Dallas, Texas, USA), CD34 (polyclonal goat CD34 antibody, Abnova, USA), CD45 (polyclonal rabbit CD45 antibody, Abnova), and KDR (monoclonal rabbit KDR antibody, Upstate), was performed on adherent mononuclear cells after 7 days of culture.

The ultrastructure of EPCs was observed by using an electron microscope. Briefly, cells were fixed with 3% glutaraldehyde at 4°C for 2 hours. After washing and postfixing with 1% O_S_O_4_ at 4°C for 30 min, the cells were washed and processed for embedding. Sections were analyzed under an electron microscope (SU-70, Hitachi).

Direct fluorescent staining was used to detect dual binding of FITC-labeled BS-lectin (Sigma) and 1,1-dioctade-cyl-3,3,3,3-tetramethylindocarbocyanine-labeled acetylated low-density lipoprotein (Dil-ac-LDL; Molecular Probe, Carlsbad, California, USA). Briefly, cells were first incubated with Dil-ac-LDL at 37°C in an atmosphere with 5% CO_2_ for 4 hours and fixed with 2% paraformaldehyde (Tianyi) for 10 minutes. After two washes, the cells were treated with BS-lectin (10 mg/L) for 2 hours. After staining, the samples were analyzed on a laser scanning confocal microscope (Leica, Wetzlar, Germany). Cells demonstrating double-positive fluorescence were identified as differentiated EPCs.

### 2.9. RNA Interference and Inhibitor/Cytokine Treatment

EPCs were grown to 70% confluency and transfected with Cav-1 siRNA or scramble siRNA as control (Santa Cruz) for 24 h or indicated times using Lipofectamine 2000 (Invitrogen). EPCs were treated with a low dose of mixed proinflammatory factors (TNF-*α*, 10 ng/mL; IL-1*β*, 5 ng/mL; IL-2, 10 ng/mL; IL-8, 10 ng/mL; and IL-12, 5 ng/mL [Peprotech EC]) for 24 h or indicated times. For PI3K and AKT inhibition, EPCs were pretreated with 4 *μ*M LY294002 (PI3K inhibitor, Calbiochem) and 2 *μ*M Wortmannin (Sigma), respectively, for 30 min, and stimulated by a mixture of proinflammatory factors for indicated times.

### 2.10. Proliferation, Migration, and Adhesion Assays

Isolated EPCs were seeded into 96-well plates at a density of 1 × 10^3^/well and incubated at 37°C. They were counted at the indicated time points to evaluate cell proliferation. In cultured EPCs, they were transfected with Cav-1 siRNA or control and seeded into a 96-well plate in triplicate at the concentration of 3 × 10^3^ cells per well. The cell growth was measured by 3-(4,5-dimethylthiazol-2-yl)-2,5-diphenyltetrazolium (MTT) bromide assay every 24 hours from day 1 to day 4. Cells were incubated with 5 mg/mL MTT for 4 h and subsequently solubilized in DMSO. The absorbance at 570 nm was then measured using a SpectraMax Plus microplate reader (Molecular Devices).

The migration ability of EPCs was measured by wound healing assay. Briefly, a total of 1 × 10^5^ isolated EPCs were seeded in 6-well plates, scratched with a sterile plastic tip, and washed with the culture medium. Subsequently, EPCs were cultured for 36 hours with complete medium containing 1% FBS. At different time points, the cells were imaged under a microscope (IX71; Olympus, Tokyo, Japan).

Fibronectin (100 *μ*g/mL) was coated onto 96-well plates for 12 hours at 37°C. EPCs at 1 × 10^4^/mL were added into the plates (1 mL/well) and allowed to attach for 30 minutes. Cell nuclei were stained with Giemsa (Dade Behring). Attached cells were counted manually in five random microscopic fields to evaluate the adhesion ability of EPCs.

### 2.11. Angiogenesis Assay

An angiogenesis assay plate (BD Pharmingen) was used to assess the angiogenetic ability of EPCs. 96-well black plates (BD Pharmingen) with clear bottom uniformly coated with BD Matrigel Matrix were incubated for 30 minutes at 37°C with 5% carbon dioxide. The EPCs were added at 1 × 10^5^/well at different time points. The samples were incubated for 24 hours at 37°C with 5% carbon dioxide. For each plate, 6.25 mL Hank's balanced saline solution (HBSS) (BD Pharmingen) was mixed with 20 *μ*L dimethyl sulfoxide (BD Pharmingen) and 50 *μ*g Calcein AM (BD Pharmingen). After incubation, the medium was carefully removed from the plates. The plates were washed by adding 100 *μ*L HBSS to each well, and the number of EPC formed tubes was counted by fluorescence microscopy (IX71; Olympus, Tokyo, Japan).

### 2.12. Real-Time Quantitative RT-PCR

Total cellular RNA was extracted from cells using a total RNA extraction miniprep system kit according to the manufacturer's instructions. RT-PCR was performed on an ABI Prism 7000 with One Step SYBR® PrimeScript™ RT-PCR Kit (TaKaRa, Shanghai, China). The primers used were the following: CAV-1, sense CTACAAGCCCAACAACAAGGC and antisense AGGAAGCTCTTGATGCACGGT; *β*-actin, sense AGCCATGTACGTAGCCATCC and antisense CTCTCAGCTGTGGTGGTGAA. RT-PCR was performed with an initial activation step of 3 min at 95°C followed by 40 cycles of 95°C for 30 s and 63°C for 30 s and a final cycle of 72°C for 7 min. Relative expression of cav1 mRNA levels was calculated using the comparative cycle threshold (CT) (2^−ΔΔCt^) method [16], and actin was used as the endogenous control to normalize the data. The expression level of the MODS group was presented as fold changes relative to the sham group at indicated time points.

### 2.13. Western Blot

EPCs were lysed using protein extract buffer (1 mL protein extract buffer with 5 *μ*L mixture of protease inhibitors (PMSF) and 5 *μ*L mixture of phosphatases) at 4°C with sonication for 30 min. Equal amounts of protein were separated on 12% SDS-PAGE and transferred onto nitrocellulose membranes. After blocking with 5% nonfat milk, the membranes were incubated with antibodies against eNOS (1 : 500, BD Bioscience, San Jose, CA, USA), p-eNOS (1 : 500, BD Bioscience), AKT (1 : 1000, Cell Signaling, Beverly, MA, USA), p-AKT (1 : 500, Cell Signaling), and *β*-actin (1 : 1000, Cell Signaling) for 12 h at 4°C. Primary antibodies were detected with horseradish peroxidase-conjugated secondary antibodies (1 : 5000), and protein bands were visualized by enhanced chemiluminescence (Amersham Biosciences, Shanghai, China). The signal intensity of phosphorylation was normalized to the corresponding total protein. Relative intensities of protein bands were analyzed with ImageJ software.

### 2.14. Measurement of NO Levels

The Griess method [17] was used to assess NO levels with a specific kit (Fluka, Shanghai, China) according to the manufacturer's instructions. The results were presented as micromole (*μ*mol) Nox of NO_3_^−^/NO_2_^−^ per liter of medium.

### 2.15. Statistical Analysis

Data are expressed as the s deviation (SD). Statistical analysis was performed by two-tailed Student's *t*-test for two groups and one way ANOVA with the Newman-Keuls post hoc test for more than two groups. *P* < 0.05 was considered statistically significant.

## 3. Results

### 3.1. The Establishment of MODS in Pigs

MODS modeling was 100% successful, and 17 out of 20 animals (85%) died during observation. A total of 15 animals (75%) developed MODS in the midanaphase of the sepsis (96-144 h after modeling). Dysfunction of 2, 3, and ≥4 organs occurred in 7, 8, and 5 cases, respectively. The incidence of MODS and mortality in the control group was 0% (*P* < 0.01).

**Figure 1:**
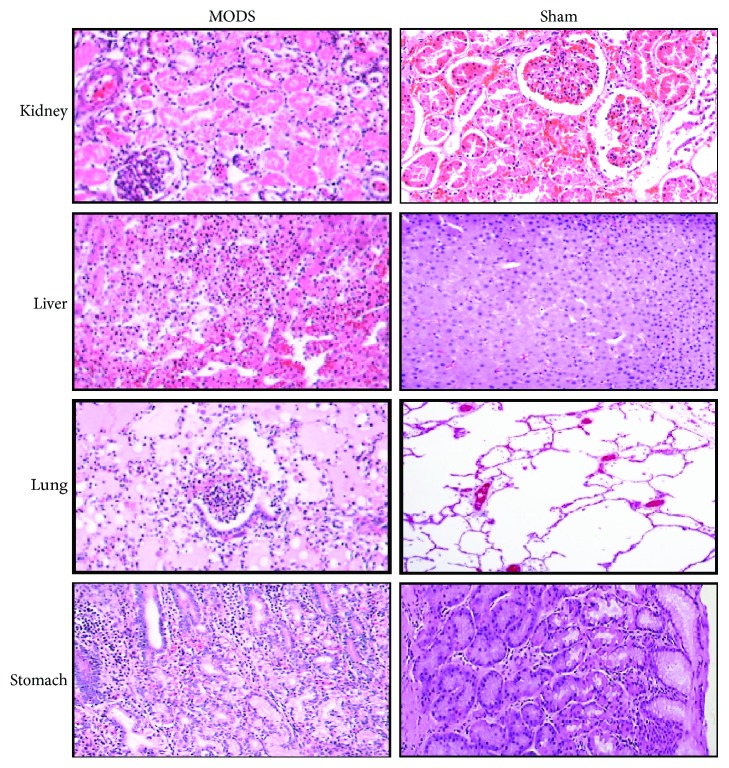
The establishment of the MODS model. Pathological changes in the kidney, liver, lung, and stomach of MODS and sham group pigs. Tissues were stained with HE and observed under a light microscope (magnification, ×200).

In the MODS group, the incidence rates of pulmonary dysfunction, cardiac dysfunction, hepatosis, renal dysfunction, and coagulation disorders were 80% (16 cases), 65% (13 cases), 55% (11 cases), 35% (7 cases), and 35% (7 cases), respectively. Pulmonary and cardiac dysfunction occurred earlier than any other organ dysfunction during sepsis (Table 1). The MODS modeling was confirmed by pathological analysis shown in Figure 1.

### 3.2. Characterization of Bone Marrow-Derived EPCs

**Figure 2:**
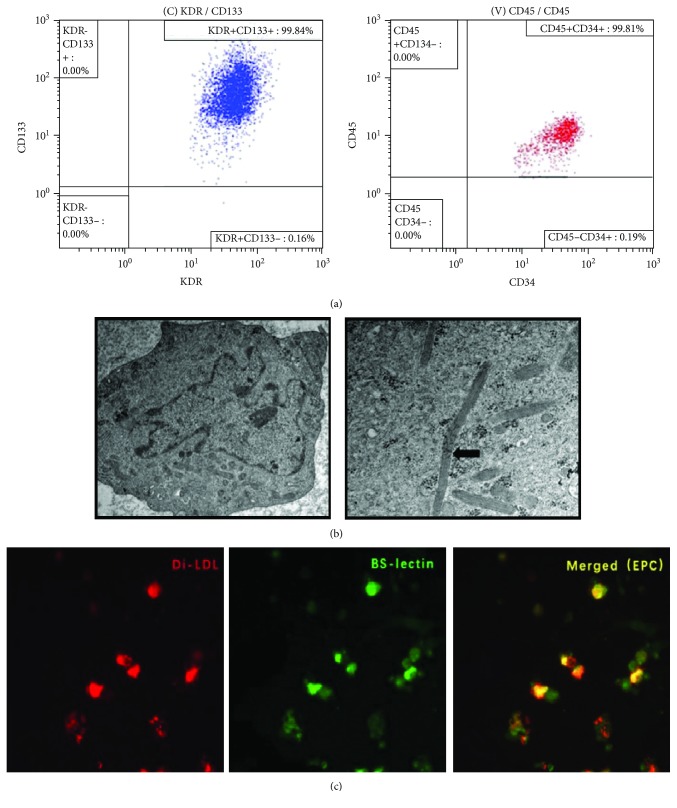
Characterization of endothelial progenitor cells (EPCs). (a) On day 7, after culture, EPCs were stained with antibodies against KDR, CD133, CD34, and CD45 and analyzed by flow cytometry. More than 99% of the cultured cells were positive for KDR, CD133, CD34, and CD45. (b) Ultrastructure of EPCs by electron microscopy. Black arrow shows a Weibel-Palade body, which is a structural characteristic of EPCs. Magnification: (left) ×5000, (right) ×30000. (c) Endothelial progenitor cells were labeled with Dil-ac-LDL and stained with FITC-labeled BS-lectin. Double-positive cells were considered EPCs. Magnification: ×400.

After 7 days of culture, flow cytometry analysis demonstrated that more than 90% of cells were positive for KDR, CD133, CD34, and CD45 (Figure 2(a)). Some rod-shaped organelles (Weibel-Palade bodies), considered a structural characteristic of EPCs, were observed in the cell plasma by transmission electron microscopy (Figure 2(b)). Furthermore, costaining assay revealed that more than 90% of adherent cells were positive for both Dil-ac-LDL uptake and BS-lectin binding (Figure 2(c)) and were recognized as EPCs.

### 3.3. In Vitro Behaviors and Cav-1 Expression of EPCs during MODS

**Figure 3:**
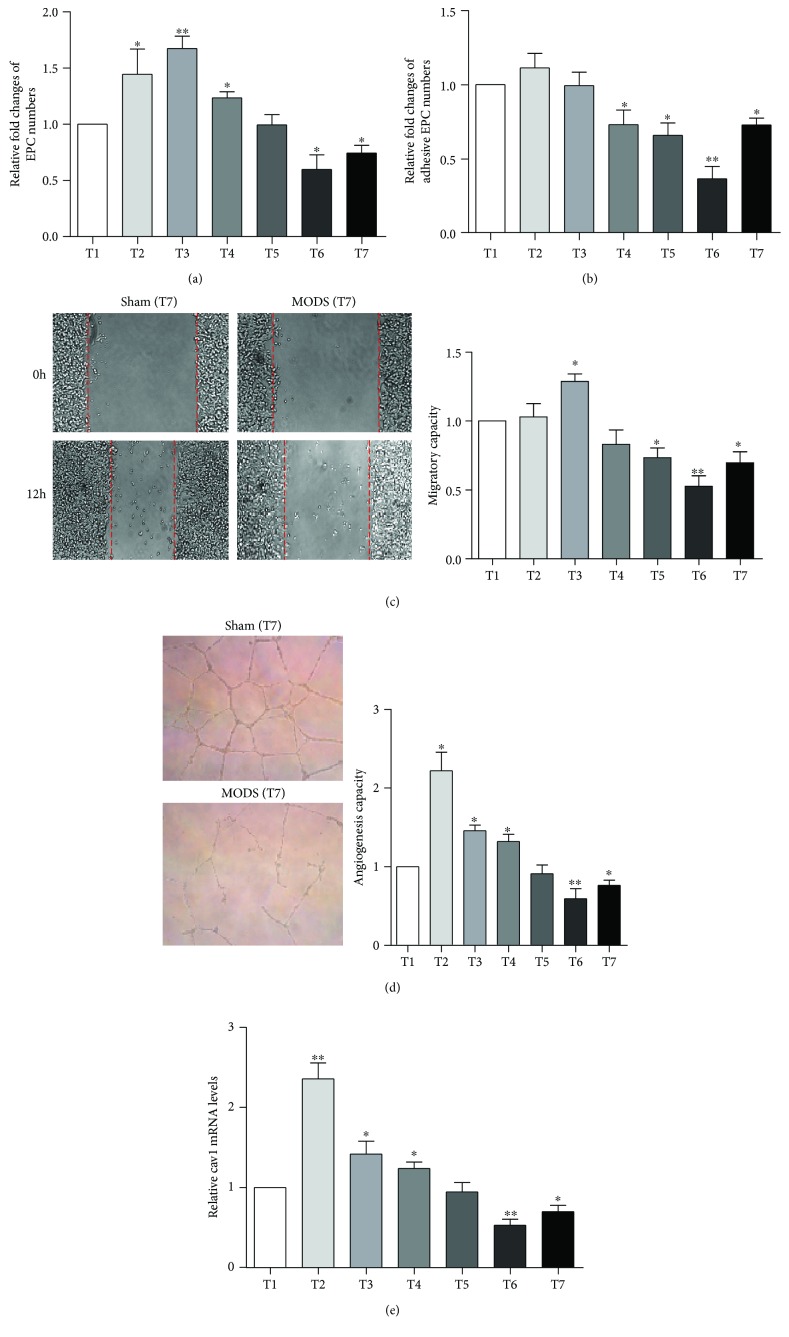
Cav-1 expression and EPC function during the progression of MODS. (a) The relative fold changes of EPC numbers in the control and MODS groups at indicated time points during MODS progression. (b) The relative fold changes of adhesive EPC numbers in the control and MODS groups at indicated time points during MODS progression. (c) Representative images of wound healing assay at indicated time points and its quantification at indicated time points during MODS progression. (d) Representative images of angiogenesis assay at indicated time points and its quantification at indicated time points during MODS progression. (e) The cav-1 mRNA levels in EPCs examined by RT-PCR. T1: 24 hours before operation; T2: 12 hours after resuscitation; T3: 24 hours after endotoxemia; T4: 72 hours after endotoxemia; T5: 96 hours after endotoxemia; T6: 144 hours after endotoxemia; and T7: 168 hours after endotoxemia. The data were presented as fold changes of the MODS group relative to the sham group. ^∗^*P* < 0.05 vs. T1.

EPCs were increased in number after hemorrhagic shock and peaked in the early phase of sepsis (24 hours after endotoxemia; T3). Then, the number of EPCs began to sharply decrease until death accompanied with the progression of MODS (Figure 3(a)). The adhesive capacities declined as MODS progressed (Figure 3(b)). The migratory capacities of EPCs reached a peak in the early phase of sepsis (T3) and then began to decline (Figure 3(c)). The angiogenic capacities of EPCs quickly increased after hemorrhagic shock to peak and remained in high level in the early phase of sepsis (T3) (Figure 3(d)). During the progression of MODS, changes of Cav-1 expression in EPCs were generally consistent with functional changes, with a rapid increase after hemorrhagic shock, and maintained at high level in the early phase of sepsis (24 hours after endotoxemia; T3). With MODS progression, Cav-1 expression levels in EPCs quickly and sharply decreased until death (Figure 3(e)). It suggests that Cav-1 expression is correlated with EPC function.

### 3.4. Caveolin-1 Expression Regulates the Function of Endothelial Progenitor Cells

**Figure 4:**
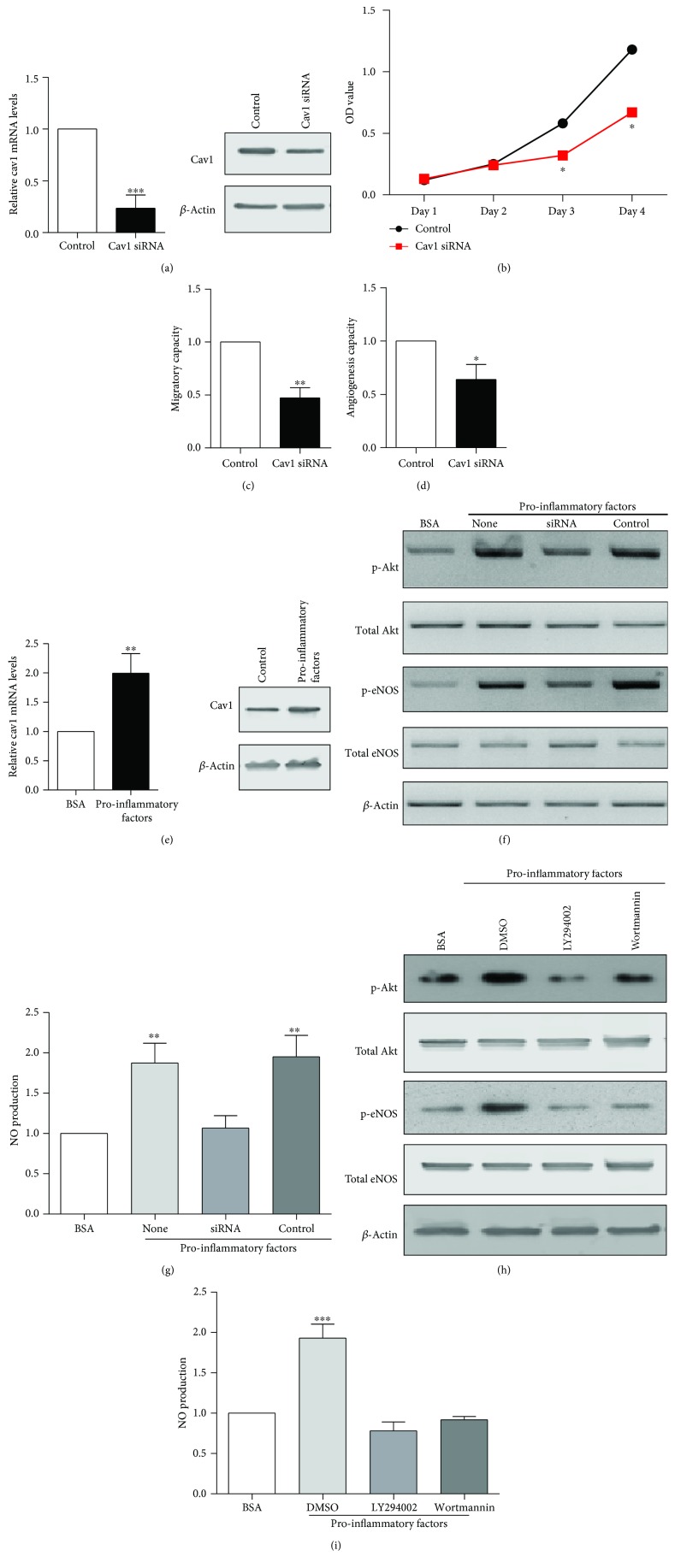
Cav-1 regulates the functions of EPCs. (a) Cav-1 mRNA and protein levels in EPCs after Cav-1 siRNA transfection for 48 h. *β*-Actin was used as internal control. (b) Cell proliferation of EPCs after Cav-1 siRNA transfection determined by MTT assay. (c) Cell migration of EPCs after Cav-1 siRNA transfection assessed by wound healing assay. (d) The angiogenic ability of EPCs after Cav-1 siRNA transfection evaluated by tube formation assay. (e) Cav-1 mRNA and protein levels in EPCs after incubation with proinflammatory factors and indicated inhibitors. *β*-Actin was used as internal control. (f) The protein expression levels of p-AKT, total AKT, p-eNOS, and total eNOS assessed by Western blot after incubation with proinflammatory factors and indicated siRNA. (g) NO levels after incubation with proinflammatory factors and indicated siRNA were detected by the Griess method. (h) The protein expression levels of p-AKT, total AKT, p-eNOS, and total eNOS assessed by Western blot after incubation with proinflammatory factors and indicated inhibitors. (i) NO levels after incubation with proinflammatory factors and indicated inhibitors were detected by the Griess method. ^∗^*P* < 0.05 vs. the control group.

To directly demonstrate that Cav-1 expression modulates EPC function, Cav-1 expression was downregulated with siRNA in cultured EPCs. In EPCs transfected with Cav-1 siRNA, Cav-1 mRNA and protein levels were greatly reduced (Figure 4(a)). In cells transfected with Cav-1 siRNA, cell proliferative (Figure 4(b)), migratory (Figure 4(c)), and angiogenic (Figure 4(d)) capacities were significantly decreased.

Based on our previous study [5, 12], we used a low dose of mixed proinflammatory factors that may simulate the early stage of sepsis *in vitro*, to induce Cav-1 expression in EPCs (Figure 4(e)). Under the treatment of mixed proinflammatory factors, p-Akt and p-eNOS were also increased (Figure 4(f)). As PI3K, the direct AKT activator, subsequently activates eNOS and increases NO secretion [13], we also measured NO production and the results show that mixed proinflammatory factors increased NO production (Figure 4(g)). Once Cav-1 was downregulated, the effects of mixed proinflammatory factors on p-Akt, p-eNOS, and NO were abolished. It suggests that mixed proinflammatory factors induced the Cav-1-activated Akt-eNOS-NO pathway. Then, we assessed whether PI3K is required for Cav-1-induced AKT and eNOS phosphorylation and NO secretion by using the PI3K inhibitors LY294002 or Wortmannin. Pretreatment of EPCs for 30 min with LY294002 (4 *μ*M) or Wortmannin (2 *μ*M) resulted in suppressed phosphorylation of AKT and eNOS (Figure 4(h)). Consistently, NO production was induced by mixed proinflammatory factors and inhibited by LY294002 and Wortmannin (Figure 4(i)). These findings suggested that Cav-1 might affect PI3K/AKT/eNOS signaling and regulate the function of EPCs.

## 4. Discussion

It is well documented that EPCs are the most important cells involved in not only epigenetic angiogenesis but also the process of organ repair by differentiation into endothelial cells *in vivo* [5]. Thus, EPCs are deemed to have potential therapeutic effects and broad clinical application prospects in the treatment of traumatic ischemic diseases and damage repair. Our previous research found that the number and function of EPCs decrease sharply during the progression of MODS [13]. However, satisfactory results were not obtained when attempting to treat MODS by autologous transplantation of EPCs [5]. In the present study, we found Cav-1 could regulate the function of EPCs likely via the PI3K/AKT/eNOS signaling pathway.

Cav-1, the major protein component of caveolae, is thought to interact, via its scaffolding domain, with numerous signaling proteins, which in turn mediate many important endothelial functions such as cell signaling, endothelial transcytosis, and endocytosis [18]. Recently, upregulation of Cav-1 has been suggested as a potential target for novel therapeutic interventions in human diseases [19, 20]. Tan et al. [7] demonstrated that Cav-1 upregulation quickly inhibits lysosomal degradation and promotes EPC proliferation via the PI3K/ERK1/2 signaling pathway. Furthermore, increasing evidence indicates that oxidative stress modulates the expression and function of Cav-1, which may improve the function of endothelial cells [21]. The current study further supported these previous findings that the number and function of EPCs might be controlled by Cav-1. In vitro treatment of EPCs with a low dose of mixed proinflammatory factors is to mimic the early stage of sepsis (T3-T4) in which Cav-1 expression in EPCs was upregulated and angiogenic capacity was enhanced compared to T1. It may represent compensatory responses in which EPCs are mobilized to maintain the homeostasis of damaged tissues and organs. However, Cav-1 expression and EPC function begin to decline when sepsis continues to progress. Thus, the protective compensatory responses fail to rescue MODS at last. Therefore, we postulated that Cav-1 upregulation could restore the repair capacity of EPCs during the progression of MODS.

There was another interesting finding in the present study. According to current knowledge, PI3K/AKT/eNOS signaling is affected by various physiological and pathological stimuli, which regulate multiple critical steps in the repair of vessel damage by EPCs [22], which was also verified in the present study. In previous findings, Cav-1 is normally involved in negative regulation of eNOS [19], while its overexpression inhibits NO production associated with eNOS activation [20]. However, in the early stage of sepsis, both Cav-1 and eNOS were upregulated *in vivo* as shown above, and the number and function of EPCs increased accordingly. In cultured EPCs, low amounts of mixed proinflammatory factors (TNF-*α*, IL-1*β*, IL-2, IL-8, and IL-12) were used in this work to confirm that the function of EPCs is likely controlled by Cav-1 through PI3K/AKT/eNOS signaling. These results might appear contradictory to the previously reported notion that Cav-1 is involved in the negative regulation of eNOS. A possible explanation is that sepsis would interfere with the normal function of Cav-1 and would occur via sepsis-induced oxidative stress, since free radicals directly affect the caveolae structure [21]. Therefore, sepsis-induced oxidative stress may alter caveolin-1 function and thus eNOS regulation [21], leading to uncontrolled activation. However, many uncertainties remain regarding the interactions of complex inflammatory factors during the phase of sepsis.

The major limitation of this study is that we used mixed inflammatory factors to upregulate Cav-1 for further investigation instead of overexpression with lentiviral vectors, for which we had technical challenges due to the relatively high molecular weight of Cav-1. It also requires in vivo studies to support that overexpression of Cav-1 in EPCs could enhance its therapeutic efficacy.

## 5. Conclusion

The current findings demonstrated that Cav-1 could regulate the function of EPCs, likely by activating the PI3K/AKT/eNOS pathway, indicating that Cav-1 could be an attractive target for the treatment of MODS.

## Figures and Tables

**(a):** 

Heart parameters	Group	T1	T2	T3	T4	T5	T6	T7
HR (time/min)	C	102 ± 10	111 ± 12	94 ± 18	89 ± 12	83±9^∗∗^	86±8^∗∗^	88±10^∗∗^
	M	118 ± 12	120 ± 13	117 ± 12	120 ± 9	128 ± 10^×^	136 ± 13^×^	142 ± 19^××^
MAP (mmHg)	C	114 ± 9	118 ± 7	119±9^∗∗^	116±8^∗∗^	112 ± 11	111 ± 12	108 ± 15
	M	112 ± 9	114 ± 12	86 ± 5^×^	92 ± 7^×^	98 ± 11	95 ± 10	94 ± 8
CO (L/min)	C	4.6 ± 1.2	4.7 ± 0.9	5.1 ± 0.9	5.6 ± 1.2	6.7±1.8^∗∗^	7.1±1.3^∗∗^	6.3±1.4^∗∗^
	M	4.8 ± 0.7	5.3 ± 1.1	7.3 ± 0.9^×^	9.6 ± 1.1^×^	13.1 ± 1.3^××^	13.0 ± 1.5^××^	13.4 ± 1.4^××^
SVRI (dyn·s/cm^5^·m^2^)	C	1246 ± 158	1183 ± 149	1050±149^∗∗^	1108±126^∗∗^	1193±134^∗∗^	1087±159^∗∗^	1060±188^∗∗^
	M	1360 ± 252	1290 ± 276	599 ± 134^××^	536 ± 111^××^	449 ± 49^××^	426 ± 51^××^	413 ± 52^××^
LVSWI (g·m/m^2^)	C	119 ± 17	121 ± 15	122 ± 15	126±14^∗∗^	131±21^∗∗^	122±18^∗∗^	116±17^∗∗^
	M	114 ± 14	119 ± 12	121 ± 10	188 ± 17^××^	211 ± 15^××^	202 ± 14^××^	203 ± 15^×^

**(b):** 

Lung parameters	Group	T1	T2	T3	T4	T5	T6	T7
MPAP (mmHg)	C	6.7 ± 1.2	6.9 ± 1.3	7.2 ± 2.4	7.8 ± 3.1	8.2 ± 4.1^∗∗^	8.0 ± 2.7^∗∗^	7.8 ± 2.6^∗∗^
	M	6.4 ± 2.0	7.1 ± 1.8	8.6 ± 2.5	12.6 ± 2.9	17.1 ± 4.4^××^	19.2 ± 3.6^××^	20.3 ± 3.9^××^
PAWP (mmHg)	C	4.6 ± 1.3	4.9 ± 1.5	5.7 ± 1.2	5.9 ± 1.3	6.2±2.5^∗∗^	6.3±2.7^∗∗^	6.5 ± 3.1
	M	4.2 ± 1.4	4.7 ± 1.8	5.5 ± 1.6	8.9 ± 2.4	11.9 ± 2.7^××^	10.1 ± 2.3^××^	7.9 ± 1.8^×^
PVRI (dyn·s/cm^5^·m^2^)	C	19.1 ± 4.3	19.2 ± 5.2	19.5 ± 5.2	19.8 ± 4.4	20.9±4.0^∗∗^	21.6±3.6^∗∗^	21.6±4.4^∗∗^
	M	18.3 ± 5.8	19.3 ± 6.4	20.0 ± 5.6	27.1 ± 4.2	28.9 ± 2.7^×^	29.8 ± 4.1^×^	36.3 ± 5.5^××^
RVSWI (g·m/m^2^)	C	6.8 ± 2.5	7.0 ± 2.4	7.5±2.2^∗∗^	8.2±4.1^∗∗^	9.1±4.8^∗∗^	9.3±3.6^∗∗^	9.7±3.6^∗∗^
	M	7.0 ± 2.7	8.8 ± 3.2	11.4 ± 2.0	21.7 ± 5.1	31.1 ± 7.6^××^	37.2 ± 6.8^××^	40.5 ± 6.9^××^
Arterial pH	C	7.46 ± 0.05	7.44 ± 0.04	7.43 ± 0.07	7.41 ± 0.09	7.40 ± 0.06	7.39 ± 0.04	7.41 ± 0.05
	M	7.44 ± 0.05	7.43 ± 0.06	7.45 ± 0.07	7.39 ± 0.1	7.35 ± 0.03	7.33 ± 0.02	7.34 ± 0.03
PaO_2_ (mmHg)	C	122.3 ± 7.6	120.1 ± 6.8	111.8±10.9^∗∗^	108.1±9.9^∗∗^	100.5±9.0^∗∗^	102.2±8.4^∗∗^	105.5±9.0^∗∗^
	M	120.2 ± 8.4	106.2 ± 9.7	81.4 ± 5.1^××^	78.3 ± 4.8^××^	71.3 ± 10.0^××^	72.6 ± 12.4^××^	73.0 ± 16.3^××^
PaCO_2_ (mmHg)	C	37.9 ± 2.4	38.4 ± 2.7	38.4±3.3^∗∗^	38.0±2.9^∗∗^	37.0±2.5^∗∗^	37.7±3.1^∗∗^	38.4±4.0^∗∗^
	M	37.8 ± 1.8	33.4 ± 2.9	31.5 ± 4.2^××^	32.3 ± 5.9^××^	33.2 ± 4.1^××^	39.1 ± 3.5^××^	47.4 ± 3.6^××^
SaO2 (%)	C	96.8 ± 1.8	94.2 ± 3.4	91.2±5.0^∗∗^	92.9±3.7^∗∗^	92.5±5.2^∗∗^	93.8±4.6^∗∗^	92.2±3.4^∗∗^
	M	97.3 ± 1.5	92.6 ± 3.4	91.4 ± 5.6	88.5 ± 7.1	85.6 ± 7.4^××^	83.2 ± 5.2^××^	80.1 ± 6.0^××^

**(c):** 

Blood RT	Group	T1	T2	T3	T4	T5	T6	T7
ALT (U/L)	C	22 ± 4	23 ± 3	23 ± 5	24 ± 4^∗^	25 ± 8^∗^	25 ± 10^∗^	26±9^∗∗^
	M	23 ± 8	24 ± 6	28 ± 10	31 ± 9^×^	36 ± 11^×^	42 ± 10^×^	43 ± 8^×^
AST (U/L)	C	48 ± 16	52 ± 16	53±15^∗∗^	54±16^∗∗^	55±20^∗∗^	51±13^∗∗^	42±21^∗∗^
	M	50 ± 22	83 ± 24	101 ± 54^××^	154 ± 77^××^	195 ± 100^××^	196 ± 92^××^	200 ± 80^××^
TB (umol/L)	C	21.9 ± 9.0	21.6 ± 7.2	22.0±5.7^∗∗^	23.2±4.4^∗∗^	22.0±11.9^∗∗^	23.5±12.3^∗∗^	24.5±8.0^∗∗^
	M	22.5 ± 13.0	26.1 ± 11.2	33.8 ± 13.5^×^	38.1 ± 12.2^×^	44.3 ± 11.5^×^	49.4 ± 15.7^×^	50.6 ± 10.0^×^
Albumin (g/L)	C	27 ± 4	26 ± 3	26 ± 5	24 ± 7	24 ± 5^∗^	25 ± 6^∗^	25 ± 3^∗^
	M	27 ± 5	26 ± 4	25 ± 6	21 ± 6	20 ± 6^×^	20 ± 5^×^	19 ± 5^×^
BUN (mmol/L)	C	2.1 ± 0.6	2.1 ± 1.1	2.2 ± 1.7	2.2 ± 1.1	2.3 ± 0.7	2.2 ± 0.5	2.2±0.6^∗∗^
	M	2.4 ± 0.5	2.5 ± 1.2	2.4 ± 1.2	2.3 ± 0.5	2.2 ± 0.6	3.9 ± 1.8	5.0 ± 3.8^××^
Cr (umol/L)	C	77 ± 11	72 ± 13	69 ± 15	65 ± 13	64 ± 16	69 ± 18	76±15^∗∗^
	M	72 ± 21	74 ± 17	68 ± 19	65 ± 18	63 ± 25	88 ± 21	107 ± 52^×^

**(d):** 

Plasma cytokines	Group	T1	T2	T3	T4	T5	T6	T7
TNF*α* (pg/mL)	C	187.3 ± 62.0	156.4 ± 54.2	123.8 ± 43.1	24 ± 4^∗^	129.0±40.2^∗∗^	130.3±34.7^∗∗^	131.1±39.5^∗∗^
	M	194.9 ± 65.8	172.5 ± 57.1	120.7 ± 55.7	31 ± 9^×^	532.4 ± 72.1^××^	465.2 ± 51.1^××^	398.9 ± 65.8^××^
IL-1*β* (pg/mL)	C	79.2 ± 7.1	72.8 ± 6.4	66.9 ± 3.9	54±16^∗∗^	63.0±4.8^∗∗^	62.7±5.7^∗∗^	59.3±6.0^∗∗^
	M	85.6 ± 18.8	71.2 ± 19.1	60.9 ± 16.4	154 ± 77^××^	325.9 ± 47.8^××^	301.4 ± 31.2^××^	249.6 ± 32.4^××^
VEGF (pg/mL)	C	17.2 ± 1.9	16.3 ± 2.9	16.9 ± 1.4	23.2±4.4^∗∗^	18.0±2.0^∗∗^	18.2±1.9^∗∗^	18.3 ± 2.1
	M	18 ± 1.5	16 ± 1.6	15.50 ± 1.2	38.1 ± 12.2^×^	26.6 ± 7.8^××^	27.1 ± 6.7^××^	29.8 ± 3.6^×^

## Data Availability

The data used to support the findings of this study are available from the corresponding author upon request.

## Abbreviations

ALT: Alanine aminotransferase
BUN: Blood urea nitrogen
Cav-1: Caveolin-1
CO: Cardiac output
Cr: Creatinine
ECs: Endothelial cells
EPCs: Endothelial progenitor cells
HBSS: Hank's balanced saline solution
MAP: Mean arterial blood pressure
MODS: Multiple organ dysfunction syndromes
PBS: Phosphate-buffered saline
TB: Total bilirubin
ULN: Upper limit of normal
VEGF: Vascular endothelial growth factor.
